# Associations of Bilateral Vestibulopathy With Cognition in Older Adults Matched With Healthy Controls for Hearing Status

**DOI:** 10.1001/jamaoto.2022.1303

**Published:** 2022-06-16

**Authors:** Joyce Bosmans, Hanne Gommeren, Griet Mertens, Patrick Cras, Sebastiaan Engelborghs, Angelique Van Ombergen, Luc Vereeck, Annick Gilles, Vincent Van Rompaey

**Affiliations:** 1 Department of Translational Neurosciences, Faculty of Medicine and Health Science, University of Antwerp, Antwerp, Belgium; 2 University Department of Otorhinolaryngology–Head and Neck Surgery, Antwerp University Hospital, Edegem, Belgium; 3 Department of Neurology, Antwerp University Hospital and Institute Born-Bunge, University of Antwerp, Antwerp, Belgium; 4 Department of Neurology, Universitair Ziekenhuis Brussel and Center for Neurosciences, Vrije Universiteit Brussel, Brussels, Belgium; 5 Department of Biomedical Sciences, University of Antwerp, Antwerp, Belgium; 6 Department of Rehabilitation Sciences and Physiotherapy, Move Antwerp, Faculty of Medicine and Health Science, University of Antwerp, Antwerp, Belgium; 7 Department of Education, Health & Social Work, University College Ghent, Ghent, Belgium

## Abstract

**Importance:**

Recent literature suggests there may be a significant effect of the vestibular system on cognition and visuospatial processing. Given the increasing prevalence of dementia and individuals at risk for it, exploring possible modifiable risk factors, including vestibular dysfunction, is vital.

**Objectives:**

To explore the association of bilateral vestibulopathy (BV) with cognitive function in older adults, taking hearing status into account, and to explore multiple vestibular characteristics and their potential associations with cognition in patients with BV.

**Design, Setting, and Participants:**

This cross-sectional study assessed older adults (age 55-84 years) with diagnosed BV from a single center using baseline measurements from the Gehoor, Evenwicht en Cognitie (GECKO) study, an ongoing prospective longitudinal cohort study. Each participant was individually matched with a healthy control based on age, sex, and hearing performance. Data were analyzed in January 2022.

**Main Outcomes and Measures:**

The primary outcome measure was cognition, measured by the Repeatable Battery for the Assessment of Neuropsychological Status for Hearing-Impaired Individuals (RBANS-H).

**Results:**

A total of 68 patients were assessed, including 34 patients with BV (mean [SD] age, 63.3 [6.0] years; 18 [53%] men) matched with 34 control individuals without BV. Overall, participants with BV had a clinically meaningful lower score on the RBANS-H total scale compared with those without BV (mean [SD] score, 98.62 [12.70] vs 105.91 [11.03]). This decline was most pronounced in the subdomains of immediate memory (mean [SD] score, 107.74 [10.66] vs 112.26 [10.66]), visuospatial cognition (mean [SD] score, 90.06 [13.34] vs 100.47 [13.91]), and attention (mean [SD] score, 94.79 [16.39] vs 102.06 [12.97]). There were no differences in language or delayed memory subdomains. Within the BV population, 1 vestibular parameter (the Performance-Oriented Mobility Assessment, in particular the balance subscale) was associated with lower cognitive scores (*r*_32_ = 0.51; 95% CI, 0.20 to 0.72; η^2^ = 0.26). Other vestibular parameters, including measurements of the peripheral vestibular end organ and questionnaires, showed no association.

**Conclusions and Relevance:**

These findings suggest there was an association between vestibular loss and cognitive impairment. Further research on the causal mechanisms underlying this association and the possible impact of vestibular rehabilitation on cognition is needed.

## Key Points

**Question:**

Is bilateral vestibulopathy (BV) associated with cognitive function in older adults?

**Findings:**

In this cross-sectional study including 34 participants with BV and 34 age-, sex-, and hearing performance–matched controls, participants with BV had worse cognitive function in general, which was most pronounced in the subdomains of immediate memory, visuospatial cognition, and attention.

**Meaning:**

These findings support existing evidence on an association between vestibular loss and cognitive impairment.

## Teaser

This cross-sectional study assesses the association of bilateral vestibulopathy with cognitive decline in older adults compared with healthy controls.

## Introduction

The peripheral vestibular end organ is located in the inner ear and codes for rotation and translation of the head. Because of its numerous projections to the central nervous system, including the brainstem, cerebellum, and widespread cortical connections, the vestibular system is involved in gaze stability, self-motion perception, orientation, navigation, and balance. An evolving body of literature suggests a significant impact of the vestibular system on cognitive function, particularly visuospatial processing. Visuospatial cognition encompasses navigation, spatial memory, mental rotation, and mental representation of 3-dimensional space.^1^

Substantial preclinical research has demonstrated long-term spatial memory deficits in animals with vestibular lesions.^2^ Vestibular damage often leads to ataxia or oscillopsia because of vestibulospinal or vestibulo-ocular reflex (VOR) deficits, respectively. Although some compensation for these reflex deficits occurs over time, spatial memory deficiencies remain.^3,4,5,6^ An important limitation in animal studies is that chemical or surgical lesions of the vestibular end organ often unintentionally also damage the cochlea. Therefore, the cognitive effects of these lesions may be due to iatrogenic hearing loss. To control for this limitation, tympanic membranes of sham control animals are often removed so that sound is no longer transmitted effectively.^2^ Nonetheless, these sham animals (intact vestibular function but removed tympanic membrane) have been observed to consistently perform better in spatial tasks than animals with vestibular lesions.^3,4,5,6^ This implies that vestibular loss, not hearing loss, is the major cause of spatial memory deficits.

These animal findings are consistent with results from human studies.^7,8,9^ Profound evidence exists that people with bilateral vestibulopathy (BV) experience impaired spatial cognition, in addition to impairments on other cognitive domains (eg, processing speed, immediate memory, and executive function).^7,8,9^ Vice versa, an overall higher prevalence of vestibular loss has been observed among people with cognitive impairment (eg, mild cognitive impairment [MCI] and Alzheimer disease [AD]).^10^ When examining brain volumetry, studies have described significant hippocampal atrophy in the BV population, with spatial memory and navigation deficits closely matching the pattern of hippocampal volume loss.^7,11^ Therefore, a direct association between hippocampal volume (a biomarker associated with AD), vestibular decline, and impaired (spatial) cognition has been observed.^7^ Hence, the question of how and to what extent vestibular loss is associated with cognitive impairment remains unanswered.

This study aims to explore the associations of BV with cognitive function, both cognition in general and different cognitive domains, in older adults. We hypothesize that individuals with BV will perform worse than healthy controls on cognition in general and on the visuospatial subdomain in particular. The secondary aim of this study is to explore multiple vestibular characteristics and their possible associations with cognition. While animal studies often discuss the possibility that concomitant hearing loss could exacerbate cognitive function decline in addition to vestibular loss, human studies investigating cognition in people with vestibular loss taking hearing status into account are limited.^8,12^ Nonetheless, the prevalence of sensorineural hearing loss in people with BV ranges from 31% to 44%.^13,14^ Therefore, this study will include hearing status when matching its control participants.

## Methods

This cross-sectional study uses data from a longitudinal study approved by the ethical committee of the University Hospital of Antwerp. The study protocol has been published previously.^15^ All participants provided written informed consent. This study is reported following the Strengthening the Reporting of Observational Studies in Epidemiology (STROBE) reporting guideline.

### Study Design

This study is a single-center, prospective, longitudinal study recruiting from November 2019 until January 2022 at the Antwerp University Hospital, Belgium. All participants were assessed during 1 baseline visit of approximately 3 hours by 2 International Conference on Harmonization Good Clinical Practice–accredited clinical researchers (J.B. and H.G.).

### Study Participants

#### Participants With BV

Participants with a diagnosis of BV according to the Bárány Society criteria^16^ were recruited from the Department of Otorhinolaryngology–Head and Neck Surgery at the Antwerp University Hospital, Belgium. Inclusion criteria for participants with BV were bilaterally reduced or absent angular VOR function documented by bilaterally pathological horizontal angular VOR gain less than 0.6 (measured by the video head impulse test [vHIT] or scleral-coil technique), reduced horizontal angular VOR gain less than 0.1 on sinusoidal stimulation on a rotatory chair (0.1 Hz, Vmax = 50°/s), or reduced caloric response (sum of bithermal maximum peak slow-phase velocity on each side <6°/s).

#### Healthy Controls

Healthy control participants were recruited from the Gehoor, Evenwicht, Cognitie (GECKO)^15^ and the Repeatable Battery for the Assessment of Neuropsychological Status for Hearing Impaired Individuals (RBANS-H) before and after cochlear implantation study.^17^ Both protocols were approved by the ethical committee of the University Hospital of Antwerp, Belgium. Each participant with BV was individually matched to a control based on age, sex, and hearing performance (best-aided speech in noise [SPIN]). All healthy controls underwent the vHIT to confirm vestibular function within reference range.

For both participants with BV and healthy controls, the following additional inclusion criteria were applied: age 55 to 84 years, Dutch as native language, no history of neurological diseases (eg, MCI or dementia), no implanted hearing aid device, and provided written informed consent. As screening for early stages of cognitive impairment may reasonably start at the age of 55 years, this age cutoff was chosen.^18^ Low education, hearing loss, obesity, smoking, and depression are risk factors associated with dementia and may negatively affect cognition.^19^ Although tinnitus has not been identified as a risk factor for dementia, research is ongoing to assess associations of tinnitus with cognition.^20^ Therefore, these variables were included in the demographic and clinical characteristics of both groups.

### Cognitive Assessment

The RBANS-H is based on the Repeatable Battery for the Assessment of Neuropsychological Status^21^ and was developed to examine cognitive function of individuals with hearing impairment.^17^ The RBANS-H is conducted by presenting an accompanying slide presentation with written explanations, which are given to support verbal instructions and to ascertain that the participant understands the instruction. In addition to visual support of the instructions, all relevant stimuli are not only presented verbally but visually as well.

The RBANS-H consists of 12 subtests: list learning, story memory, figure copy, line orientation, picture naming, semantic fluency, digit span, coding, list recall, list recognition, story recall, and figure recall. It measures the cognitive domains of immediate memory, visuospatial, language, attention, and delayed memory. Total scores of the subtests are converted into index scores per cognitive domain. These index scores are normed based on the age of the participant. The sum of all index scores is used to determine the RBANS-H total scaled score (following the distribution of the Wechsler IQ scale, 100 [SD, 15]).^22^ A scaled score (index score for each cognitive domain or RBANS-H total scaled score) of 85 or lower indicates a lower-than-expected cognitive result. Therefore, the cutoff of 85 is used for cognitive impairment.^23,24^

### Vestibular Assessment

#### Video Head Impulse Test

The vHIT is a vestibular test measuring semi-circular canal function and the VOR. For this study, we used ICS Impulse (Natus). Participants are instructed to focus on a fixation dot at eye level 1 m in front of them. They experience short, high-velocity head thrusts in the direction of all 6 (lateral, superior, and posterior; left and right ear) semicircular canals.

#### Cervical Vestibular–Evoked Myogenic Potentials

Cervical vestibular–evoked myogenic potentials are ipsilateral inhibiting muscle potentials measured at the level of the contracted sternocleidomastoid (SCM) muscle using the validated Neuro-Audio device with electromyography feedback (Neurosoft). Short tone bursts presented through insert-earphones evoke these potentials. Participants lie in a supine position and are instructed to lift and rotate their head to 1 side, thus tensioning the SCM muscle, while stimuli are presented in the contralateral ear. A typical cervical vestibular–evoked myogenic potential is biphasic and characterized by 2 distinctive peaks (p13, n23). Normative ranges are applied.^25^

#### Rotatory Chair Testing

Rotatory chair testing is a midfrequency test of the lateral semicircular canals testing a range of different frequencies, from 0.01 Hz to 0.64 Hz. Participants are tested with their eyes closed by means of electronystagmography. The sinusoid method oscillates the chair in yaw at varying frequencies, typically with peak velocities that remain constant at 50 or 60 °/s.^26^

#### Caloric Irrigation

Bithermal caloric irrigation (33 °C/44 °C) stimulates each lateral semicircular canal independently. It can identify the extent to which the vestibular system is responsive and how symmetric the responses are, between left and right.

### Clinical Balance Testing

The Timed Up-and-Go (TUG) balance test evaluates mobility, fall risk, balance, and walking pattern.^27^ Participants start in a seated position on a chair. They are then instructed to stand up, walk 3 m, turn around, walk back to the chair, and sit down again. This task is repeated 3 times, and the mean time needed to complete this task is measured.

The Performance-Oriented Mobility Assessment (POMA) test consists of 2 parts.^28,29^ The first test evaluates balance abilities in a chair and while standing (POMA-B). The other part assesses dynamic balance during gait on an even walkway (POMA-G).^28,29^

In Belgium according to the National Institute for Health and Disability Insurance (RIZIV), to obtain reimbursement of gait rehabilitation (age ≥65 years), an increased risk of falling needs to be objectified. This is done by performing the TUG (completion time, >20 seconds), POMA (total score, <20/28), and/or the timed chair stands (score, >14 seconds). Based on these criteria, the TUG and POMA are included in current study.

The Functional Gate Assessment is used to evaluate postural stability and balance during walking tasks. Participants are asked to cross a 6 m long walkway, each time with a different instruction, including: changing walking speed, walking with horizontal head turns, walking with vertical head turns, performing a 180° turn, stepping over an obstacle, walking with arms folded across the chest and with feet aligned heel to toe in tandem, walking with eyes closed, walking backward, and walking up and down stairs.^30,31,32,33^

Additionally, we assessed 3 patient-reported outcomes: dizziness, balance confidence, and oscillopsia. The Dizziness Handicap Inventory questionnaire quantifies self-perceived handicap resulting from dizziness and unsteadiness due to vestibular system diseases.^34^ Balance confidence was evaluated using the Short Falls Efficacy Scale International. Seven activities of daily living commonly performed by older adults are presented and are scored based on their concern about falling.^35^ The Oscillopsia Severity Questionnaire assesses the feeling of oscillopsia while performing different activities or engaging in different situations.^36,37^

### Hearing Assessment

Unaided pure-tone audiometry was performed to calculate the Fletcher Index high (mean threshold of 1000 Hz, 2000 Hz, and 4000 Hz).^38^ In addition to the pure-tone audiometry, speech audiometry in noise was evaluated by the Leuven Intelligibility Sentences Test using an adaptive procedure.^39^ The SPIN test is conducted in free field using a loudspeaker at a distance of 1 m at 0° azimuth. The frequency spectrum of the noise matches the long-term average frequency spectrum of the speech signal. The noise level is constant at 65 dB sound pressure level (SPL), while the speech level is adapted according to the response of the patient. A correct repetition of the keywords of a sentence decreases speech level of 2 dB SPL, while an incorrect response increases speech level of 2 dB SPL. Each list comprises 10 sentences and two lists are conducted to acquire the speech reception threshold. This threshold is calculated by calculating the mean speech levels of the last 5 sentences of the last list and the imaginary 11th sentence. To most closely resemble real-world hearing status, the best-aided SPIN results were used.

### Statistical Analysis

All data were stored in OpenClinica electronic data capture software. For all statistical analyses, JMP Pro statistical software version 15 (JMP Statistical Discovery) was used.

To obtain an estimation of the sample size needed to detect significant differences in the primary outcome variable, RBANS-H total score, a 2-tailed paired *t* test was performed on previously collected in-house validation data of the RBANS-H. The proposed sample size is 34 participants per group, which holds a power of 80% to detect a minimum clinically important difference of 4 with an estimated SD of differences of 8 and a significance level of α = .05.

The objectives of this study were to evaluate cognition (in general and the different cognitive domains) of participants with BV in comparison with healthy controls and to determine whether cognition was correlated with vestibular characteristics. The Levene test was used to evaluate the assumption of equal variances. Normality was determined by visualizing the data in histograms. Equal variances and the normality of the reported data were confirmed. Therefore, parametric tests with the mean and SD of the variables are reported. For the first objective, RBANS-H subtest index scores and the total scale score were compared between matched participants with BV and healthy controls using paired effect size metrics. Effect sizes (Cohen *d*) and 95% CIs are presented, with *d* of 0.2 indicating a small effect; 0.5, a medium effect; and 0.8, a large effect.^40^ For the second objective, associations between different vestibular variables and cognition within the BV population were explored by performing the η^2^ effect size metric, with η^2^ of 0.01 indicating a small effect; 0.06, a medium effect; and 0.14, a large effect.^40^ Data were analyzed in January 2022.

## Results

### Study Population

A total of 68 individuals were assessed, including 34 participants with BV (mean [SD] age, 63.3 [6.0] years; 18 [53%] men) matched with 34 healthy controls. The groups did not differ in the relevant demographic and clinical characteristics in any clinically meaningful way (Table 1).

**Table 1.** Demographic Characteristics of People With Bilateral Vestibulopathy and Healthy Controls

Characteristic	Participants, No. (%)	
	Bilateral vestibulopathy (n = 34)	Healthy controls (n = 34)
Sex		
Men	18 (52.9)	18 (52.9)
Women	16 (47.1)	16 (47.1)
Age, mean (SD), y	63.3 (6.0)	63.5 (5.7)
Hearing level		
FI_high,_ best ear unaided, mean (SD), dB HL	45.4 (22.6)	39.0 (22.4)
SPIN, best aided SRT, mean (SD)	−0.01 (5.7)	−1.7 (3.3)
Hearing aid ownership	16/17/1	14/20/0
Yes	16 (47.1)	14 (41.2)
No	17 (50.0)	20 (58.4)
NA	1 (2.9)	0
Education level, mean (SD), y^a^	13.3 (3.4)	14.3 (2.2)
BMI, mean (SD)	26.8 (3.8)	25.7 (4.1)
Smoking		
Yes	5 (14.7)	2 (5.9)
No	25 (73.5)	31 (91.2)
NA	4 (11.8)	1 (2.9)
Tinnitus		
Yes	11 (32.4)	13 (54.2)
No	5 (14.7)	9 (26.5)
NA	18 (52.9)	12 35.3)
Depression, mean (SD)^b^	8.9 (7.0)	7.5 (7.1)

Abbreviations: BMI, body mass index (calculated as weight in kilograms divided by height in meters squared); dB HL, decibel hearing level; FI_high_, Fletcher index high (mean 1, 2, and 4 kHz); NA, not available; SPIN, speech-in-noise; SRT, speech reception threshold.

^a^ Education level indicates the number of years spent in school, starting from the age of 6 years old.

^b^ Measured using Beck Depression Inventory.

To confirm the diagnosis of BV, the Bárány Society criteria needed to be met.^16^ All 3 criteria (bilaterally reduced response of vHIT, rotatory chair test, and caloric testing) were met by 13 individuals (38.2%) with vestibular loss. In 11 individuals (32.4%), 2 of 3 criteria were fulfilled, and the remaining 10 individuals (29.4%) met 1 criterion. Of all 34 individuals with BV, 11 individuals had a variation in the *COCH* gene causing DFNA9, an autosomal dominant disorder causing both progressive hearing loss and bilateral vestibular loss.^41,42^

### Vestibular Loss and Cognitive Performance

All 34 participants with BV and their matched healthy controls completed the RBANS-H. Overall, participants with BV obtained a clinically meaningful lower score on the RBANS-H compared with health controls (mean [SD] total score, 98.62 [12.70] vs 105.91 [11.03]; difference, 7.3; 95% CI, 0.14 to 1.22; *d* = 0.68). Participants with BV scored worse than healthy controls on all subscales (Figure). The magnitudes of the difference in performance between BV and healthy controls were greatest on visuospatial cognition (mean [SD] score, 90.06 [13.34] vs 100.47 [13.91]; *d* = 0.98), attention (mean [SD] score, 94.79 [16.39] vs 102.06 [12.97]; *d* = 0.68), and immediate memory (mean [SD] score, 107.74 [10.66] vs 112.26 [10.66]; *d* = 0.42) (Table 2).

**Figure.**
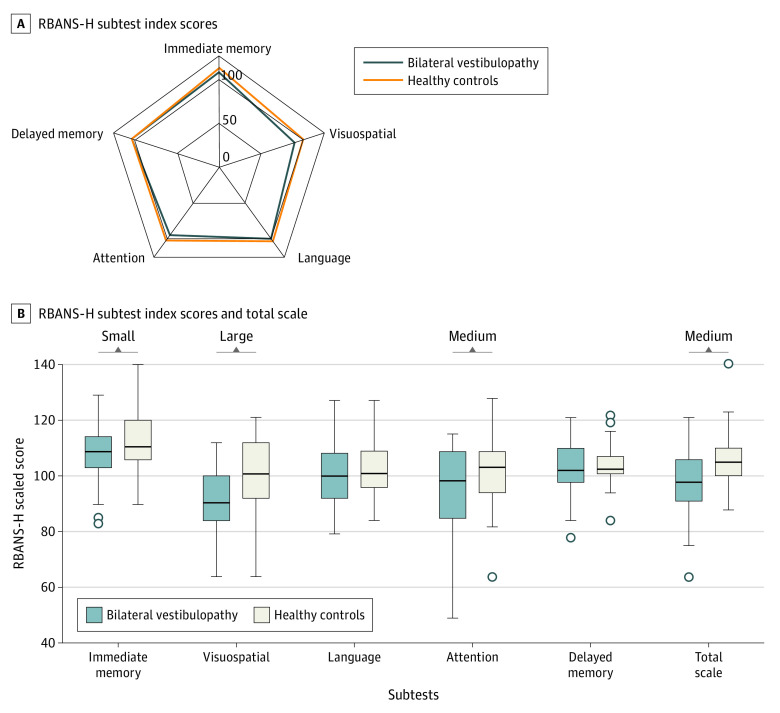
Comparison of Repeatable Battery for the Assessment of Neuropsychological Status for Hearing-Impaired Individuals (RBANS-H) Scores Between Individuals With Bilateral Vestibulopathy and Their Matched Healthy Controls B, Whiskers indicate range; boxes, IQR; bold line, median. Small, medium, and large indicate clinically meaningful Cohen *d* effect sizes.

**Table 2.** Results of the RBANS-H Total and Index Scores Including Effect Size in People With BV and Their Matched HC

RBANS-H index score	Mean (SD)		Cohen *d*^a^	Interpretation effect
	BV	HC		
Immediate memory	107.74 (10.66)	112.26 (10.66)	.42	Small
Visuospatial	90.06 (13.34)	100.47 (13.91)	.98	Large
Language	100.41 (11.82)	103.35 (9.95)	.28	Small
Attention	94.79 (16.39)	102.06 (12.97)	.68	Medium
Delayed memory	103.56 (9.68)	104.32 (7.19)	.07	Trivial
Total scale	98.62 (12.70)	105.91 (11.03)	.68	Medium

Abbreviations: BV, bilateral vestibulopathy; HC, healthy control; RBANS-H, Repeatable Battery for the Assessment of Neuropsychological Status for Hearing-impaired individuals.

^a^ As data are paired, the Cohen *d* resembles the effect size for the sum of the magnitude of the difference in each of the RBANS-H index paired scores.

### Vestibular Characteristics and Cognition

Despite results showing worse cognitive scores in participants with BV, it remains unknown which vestibular characteristics could explain this pattern of cognitive loss. Possible vestibular characteristics include measurements of the peripheral vestibular end organ, clinical balance testing, and questionnaires documenting self-perceived handicap, balance confidence, and oscillopsia. An overview can be found in Table 3. These analyses only include data of participants with BV, excluding healthy controls.

**Table 3.** Vestibular Characteristics and Association With the RBANS-H Total Scale Scores

Test	Outcome	η^2^	Interpretation effect
**Peripheral vestibular end organ: sacculus**			
Presence of intact C-VEMP responses, No.			
0	21^a^	.05	Small
1	11^a^		
Both	2^a^		
**Peripheral vestibular end organ: lateral semicircular canals**			
VOR gain, mean (SD)			
vHIT, mean (SD)^b^	0.39 (0.33)	.01	Small
Rotatory chair	0.15 (0.18)	.05	Small
Caloric response: bithermal maximum peak slow-phase velocity on each side, mean (SD)^b^	2.80 (2.71)	.01	Small
**Peripheral vestibular end organ**			
Bárány criteria met, No.			
1	10^a^	.01	Trivial
2	11^a^		
3	13^a^		
**Clinical balance testing**			
TUG score, mean (SD)	9.13 (2.20)	.03	Small
POMA total score, mean (SD)	25.15 (4.10)	.20	Large
FGA total score, mean (SD)	18.09 (6.88)	.02	Small
**Questionnaires**			
DHI total score, mean (SD)	37.19 (24.37)	<.01	Trivial
Short FES-I total score, mean (SD)	13.72 (5.48)	.01	Trivial
OSQ score mean (SD)	2.74 (1.02)	.02	Small

Abbreviations: C-VEMP, cervical vestibular evoked myogenic potentials; DHI, Dizziness Handicap Inventory; FGA, Functional Gait Assessment; OSQ, Oscillopsia Severity Questionnaire; POMA, Performance-Oriented Mobility Assessment; Short FES-I, Short Falls Efficacy Scale International; TUG, Timed Up-and-Go; vHIT, Video Head Impulse Test; VOR, vestibulo-ocular reflex.

^a^ Presented as number of participants in category.

^b^ For the vHIT and caloric response, each person had 2 values (left and right lateral VOR gain and sum of bithermal maximum peak slow-phase velocity per ear, respectively). For the analyses of these variables, each side/ear was analyzed, resulting in 68 ears (instead of 34 people).

Measurements of the peripheral vestibular end organ and questionnaires included in this study demonstrated no clinically meaningful association with RBANS-H total scores. Regarding clinical balance assessments, only the POMA demonstrated a clinically meaningful positive correlation with RBANS-H total scores (*r*_32_ = 0.45; 95% CI, 0.13 to 0.68; η^2^ = 0.20). Here, more impaired POMA scores indicate worse balance ability and are associated with worse RBANS-H total scores. Since the POMA test includes 2 subscales (balance and gait), correlations for both subscales were calculated. Results indicated that the balance subscale (*r*_32_ = 0.51; 95% CI, 0.20 to 0.72; η^2^ = 0.26) but not the gait subscale (*r*_32_ = 0.15; 95% CI, −0.19 to 0.47; η^2^ = 0.02) demonstrated a clinically meaningful positive correlation with the RBANS-H total scores.

To explore possible associations of demographic, psychological, and hearing covariates with this correlation, a general linear model analysis was performed. No significant associations of sex, age, years of education, anxiety (measured by the Hospital Anxiety and Depression Scale), depression (measured by the Hospital Anxiety and Depression Scale and Beck Depression Inventory), or hearing (best-aided SPIN and unaided Fletcher Index high: best ear) were observed.

## Discussion

The primary aim of this cross-sectional study was to investigate the association of BV with cognitive function in older adults. Overall, participants with BV had a clinically meaningful lower score on the RBANS-H total scale, indicating a cognitive decline in general. This cognitive decline was most pronounced in the subdomains of immediate memory, visuospatial cognition, and attention. Moreover, even though no participant had a history of neurological disease, 3 participants with BV had a total score worse than expected when compared with a normative group (total scaled score ≤85), which could be indicative of cognitive impairment. As the RBANS-H is a cognitive screening test and more information is needed to make a formal diagnosis, a referral to the neurology department or memory clinic was suggested to closely monitor progressive cognitive decline. Despite animal studies emphasizing the importance of accounting for possible concomitant hearing loss, human studies, including data or corrections for hearing status, are often lacking.^8,43^ As such, this study included hearing status in the individual matching procedure.

Our secondary aim was to explore vestibular characteristics and their potential associations with cognitive performance in participants with BV. A 2020 study by Pineault et al^44^ found bilateral saccular and semicircular canal vestibular impairments were associated with impairment of various domains of cognition. Surprisingly, we were unable to detect an association between measurements of the peripheral vestibular end organ (including saccular and semicircular canal measurements) and cognition. However, this claim cannot be made unambiguously as, in our study, most participants with BV met more than 1 criterion of the Bárány Society. Therefore, an interaction effect between saccule and semicircular canal dysfunction may be present. On the other hand, 1 clinical balance assessment (the POMA, balance subscale) was the only vestibular assessment parameter associated with cognition in BV in a clinically meaningful way. Previous literature on the association between the POMA and cognitive function found that older adults at risk for falls had reduced cognitive function scores, supporting our findings.^9,45,46,47^ However, studies are limited, and future studies examining the predictive value of the POMA (and its balance subscale) in cognitive decline are warranted. Furthermore, no clinically meaningful associations of demographic, psychological, or hearing status covariates were observed. This supports evidence of a link between vestibular dysfunction and cognitive impairment, regardless of hearing status.

### Vestibular Loss as a Risk Factor for AD

There is evolving evidence of an association between vestibular loss and cognitive impairment, in particular AD.^1,7,43,48,49^ From a neuropsychological point of view, the cognitive profile of BV and the early stages of AD appear to overlap. Of particular interest is a comparison with MCI. People with MCI are characterized by cognitive impairment out of proportion to the age and educational level of the individual, without impeding activities of daily life. Different subtypes of MCI exist, where amnestic MCI has the highest conversion rate to AD (annual conversion rate, 12%-15%) and is therefore considered the most relevant group to compare with people with BV.^50,51^

As the nomenclature implies, people with amnestic MCI demonstrate a prominent episodic memory loss, in which they have difficulty with learning and retaining new information. After a delay interval, people with amnestic MCI have trouble with free recall tasks. Moreover, people with amnestic MCI who convert to AD also struggle with a recognition task, whereas people who do not convert benefit from these cues.^52,53^ In this study, participants with BV also demonstrated episodic memory loss, but their delayed memory remained intact. Other patterns of cognitive impairment, such as visuospatial loss, may still be consistent with underlying AD pathological mechanisms and may therefore be observed in amnestic MCI.^24^ Regarding language, impairment is atypical and often only present in more severe AD. Taken together, the visuospatial and language patterns in amnestic MCI agree with the cognitive patterns of BV. Finally, people with amnestic MCI often have mild problems performing complex functional tasks (eg, planning, finances, cooking). They may be less efficient, make more mistakes, and take longer to finish a task, but they are still able to do so independently.^24^ People with BV demonstrated difficulties in the attention subdomain, which encompasses executive function.^54^ Therefore, people with BV may struggle with planning and performing complex functional tasks, generally matching the cognitive profile of amnestic MCI. In summary, an overlap between the cognitive profile of BV and amnestic MCI can be found (Table 4). These results further support and extend evidence of an association between vestibular loss and cognitive impairment, in particular AD.

**Table 4.** Cognitive Profile of Individuals With BV and Amnestic MCI

Cognitive domain	BV	Amnestic MCI
Immediate memory	Impaired	Impaired
Delayed memory	Preserved	Impaired
Visuospatial	Impaired	Impairments possible
Language	Preserved	Atypical but possible
Attention	Impaired	Mild problems performing complex functional tasks

Abbreviations: BV, bilateral vestibulopathy; MCI, mild cognitive impairment.

Although the association between vestibular loss and cognitive impairment is gaining evidence, a causal relationship has not been established. Several causal theories have been proposed to explain the link between dementia and hearing loss, the largest modifiable risk factor for dementia.^19,55,56^ These theories can also be applied to vestibular loss being a modifiable risk factor for dementia and, in particular, AD. A first hypothesis is the cognitive load hypothesis, which states that cognitive resources are diverted to maintain balance at the expense of other cognitive processes.^1^ Second, the deprivation hypothesis or cascade hypothesis describes that vestibular loss, and therefore reduced vestibular input, leads to accelerated brain atrophy, which then leads to dementia. Previously observed hippocampal atrophy (a biomarker of AD) in people with vestibular loss strengthens this hypothesis.^7,57^ A third hypothesis focuses more on psychosocial aspects of vestibular loss, namely the social isolation hypothesis. People with vestibular loss often experience living in fear and anxiety because they feel unsafe and fearful of falling. Therefore, they restrict their participation in activities and travel, leading to living a more socially isolated life.^58,59^ Finally, the common cause hypothesis assumes that both vestibular loss and cognitive impairment are the result of a common neurodegenerative process. Both vestibular loss and dementia are heterogeneous and involve many factors. Risk factors may vary and coexist.^49^ The exploration and identification of causal mechanisms underlying the association between vestibular loss and cognitive impairment may allow for early detection of people at risk for AD. Furthermore, identification may prevent or slow down progression along the AD continuum through proper treatment of vestibular loss.

### Treating Vestibular Loss

Given the association between vestibular function and cognition, one may hypothesize that restoration of vestibular function (by either vestibular rehabilitation, vestibular implant, or vibrotactile feedback) may increase or preserve cognition.^60,61,62,63,64^ A study by Sugaya et al^65^ trained 60 people with intractable dizziness to perform a 30-minute vestibular rehabilitation program by themselves. They demonstrated a significant improvement in cognition (visuospatial cognition, attention, and executive function, as measured by the Trail Making Test).^65^ Furthermore, vestibular rehabilitation should encompass balance exercises and training people to not stop walking while talking. Improvement in balance performance will decrease the cognitive load associated with balance performance itself. More studies examining the effect of restoration of vestibular function on cognition (including >1 cognitive test) in a population with vestibular loss are warranted. This may provide interesting results in future research, including potential treatment options within dementia research.

### Limitations

This study has some limitations. The inclusion of 34 participants per group was the lower limit of the proposed sample size. Although this number is estimated to be sufficient, a larger sample size would benefit the analysis and interpretation of results. A second limitation is the use of the RBANS-H as the neuropsychological assessment. This test is able to provide an estimation of total cognition and multiple relevant cognitive domains while only taking 30 minutes to administer. However, an extended formal neuropsychological evaluation would be beneficial to obtain a more in-depth assessment of cognition and would be able to detect more subtle differences. In addition, the RBANS-H is normed based on age, whereas neuropsychological tests, which are also normed based on sex and education level, would be preferable.

## Conclusions

This cross-sectional study found that individuals with BV demonstrated cognitive deficits compared with healthy controls. These deficits were most pronounced in the cognitive subdomains of immediate memory, visuospatial cognition, and attention. However, language and delayed memory subdomains remained preserved. This cognitive loss was found to be independent of concurrent hearing loss. In individuals with BV, cognitive deficits were associated with 1 clinical balance assessment, namely the POMA, and more specifically, the balance subscale. Other vestibular parameters, such as measurements of the peripheral vestibular end organ and questionnaires, showed no clinically meaningful association. These results support and extend previous literature on an association between vestibular loss and cognitive impairment, in particular AD. However, further research investigating causal mechanisms and the impact of vestibular treatment on cognition is recommended.
